# hiPSC‐derived iMSCs: NextGen MSCs as an advanced therapeutically active cell resource for regenerative medicine

**DOI:** 10.1111/jcmm.12839

**Published:** 2016-04-21

**Authors:** Vikram Sabapathy, Sanjay Kumar

**Affiliations:** ^1^ Center for Stem Cell ResearchA Unit of inStem BengaluruChristian Medical CollegeVelloreTamil NaduIndia

**Keywords:** mesenchymal stem cells, MSCs, induced pluripotent stem cells, hiPSCs, iMSCs

## Abstract

Mesenchymal stem cells (MSCs) are being assessed for ameliorating the severity of graft‐versus‐host disease, autoimmune conditions, musculoskeletal injuries and cardiovascular diseases. While most of these clinical therapeutic applications require substantial cell quantities, the number of MSCs that can be obtained initially from a single donor remains limited. The utility of MSCs derived from human‐induced pluripotent stem cells (hiPSCs) has been shown in recent pre‐clinical studies. Since adult MSCs have limited capability regarding proliferation, the quantum of bioactive factor secretion and immunomodulation ability may be constrained. Hence, the alternate source of MSCs is being considered to replace the commonly used adult tissue‐derived MSCs. The MSCs have been obtained from various adult and foetal tissues. The hiPSC‐derived MSCs (iMSCs) are transpiring as an attractive source of MSCs because during reprogramming process, cells undergo rejuvination, exhibiting better cellular vitality such as survival, proliferation and differentiations potentials. The autologous iMSCs could be considered as an inexhaustible source of MSCs that could be used to meet the unmet clinical needs. Human‐induced PSC‐derived MSCs are reported to be superior when compared to the adult MSCs regarding cell proliferation, immunomodulation, cytokines profiles, microenvironment modulating exosomes and bioactive paracrine factors secretion. Strategies such as derivation and propagation of iMSCs in chemically defined culture conditions and use of footprint‐free safer reprogramming strategies have contributed towards the development of clinically relevant cell types. In this review, the role of iPSC‐derived mesenchymal stromal cells (iMSCs) as an alternate source of therapeutically active MSCs has been described. Additionally, we also describe the role of iMSCs in regenerative medical applications, the necessary strategies, and the regulatory policies that have to be enforced to render iMSC's effectiveness in translational medicine.

## Introduction

Mesenchymal stromal cells (MSCs) are assorted cell preparations and only a rare subpopulation often referred to as ‘mesenchymal stem cells’ retains clonogenic proliferation ability & multilineage differentiation potential 1. Mesenchymal stem cell preparations are significantly affected by starting cell source/material, such as bone marrow (BM), adipose tissue (AT) or other adult/perinatal tissue source; cell culture surface, media composition and other *in vitro* tissue culture conditions 1, 2, 9. Furthermore, they acquire phenotypic, biochemical, molecular as well as functional changes during long‐term *in vitro* culture expansion ending in replicative senescence 7, 8. So far, MSCs are occasionally defined by their plastic adherent growth displaying fibroblast‐like cellular colonies, a panel of positive (CD73, CD90, CD105) and negative cell surface markers (CD11b/CD14, CD34, CD45, CD79α/CD19) for phenotypic characterization and their capacity to differentiate towards at least trilineage differentiations such as adipogenic, osteogenic and chondrogenic lineages 2, 4. Many researchers indicate that plasticity and immunomodulatory capabilities of the MSCs contribute towards unique therapeutic potentials of the MSCs 1, 2. The bone marrow MSCs (BMMSCs) are considered to be the gold standard in the field of MSCs. However, their invasive accessibility and lower proliferation potential significantly undermine their ability to be considered for mainstream therapeutic applications 3. The therapeutic potency of MSCs is often limited because of age or pathologically related impairments regarding cell survival, proliferation and differentiations potential of BMMSCs 4, 5, 6. Before adult MSCs can exert its therapeutic potential *in vivo*; we must determine reasons behind their limited proliferation capability, quick down‐gradation of their differentiation potentials and secrete minimal protective factors during their expansion *ex vivo*? 7, 8 The adult MSCs unveil time‐limited functions under both *in vivo* and *in vitro* conditions 9, 10. Exploration for an alternate source of MSCs resulted in several groups reporting successful isolation of MSCs like cells from foetal, neonatal 11, 12, 13, 14, 15 and embryonic stem cells (ESCs) 16, 17, 18. As a result of the current deficit in adult MSCs regarding inadequacy in MSCs passages, cell numbers and consistencies in cellular behaviour; alternative, easily accessible, safe and healthy populations of MSCs are being considered for clinical applications 3. The iPSC‐derived MSCs (iMSCs) are emerging as an attractive option for obtaining a substantial population of stem cells in a sustained manner for regenerative medical applications 3. The achievement of cell‐based therapy of MSCs in preclinical trials has precipitated success in human translational applications 19.

## Therapeutically active MSCs derived from human bone marrow

In the field of regenerative medicine, human mesenchymal stem cells (hMSCs) have transpired to be a promising candidate. Bone marrow‐derived MSCs (BMMSCs) have been used as a predominant source of MSCs. Bone marrow‐derived MSCs have been successfully used in a significant number of clinical and pre‐clinical applications 20, 21. In the early 20th century, Maximow and Friedenstein were the first to investigate the role of bone marrow fibroblast‐like subset cells in maintaining the haematopoiesis 22. The BMMSCs were first isolated and propagated under *in vitro* culture conditions in 1970s by Friedenstein *et al*. 21, 22. In 1991, Arnold Caplan termed the cells MSCs based on the ability of the cells to give rise to distinct tissue lineages 23. Maureen Owen further characterized the MSCs and observed the heterogeneity in its population 21, 24. The *in vivo* administration of the MSCs in animal and humans has shown to be safe without triggering adverse immune reaction or any tumour formation 25. Subsequently, MSCs have been shown to modulate the immune response and prevent graft‐versus‐host disease (GVHD) 26, 27. Above all MSCs has been demonstrated to be effective in both pre‐clinical and clinical stages in orthopaedic applications, cardiovascular therapies, burns, wounds, ulcers, neurodegenerative disorders, spinal cord injury, autoimmune disorders, *etc*., 28. Mesenchymal stem cells exert their biological functions through cellular migration, local engraftment, self‐renewal, plasticity, and secretion of various bioactive compounds. These intrinsic characteristics render the MSCs ideal for regenerative medical applications 29. Moreover, MSCs can be engineered to secrete various bioactive factors through viral or non‐viral‐based methods, which enhance the capabilities of MSCs in therapeutic applications 30, 31. The low proliferation potential of the adult BMMSCs renders the BMMSCs unsuitable for clinical applications 32. Further, the limited accessibility, difficulty in obtaining patient consent and invasive procedure contribute towards un‐usability of BMMSCs for routine clinical treatment. During specific times extraction of autologous MSCs from the patients might be counter‐productive for the management of the patients 3. The BMMSCs are short‐lived and hence cannot ensure consistent, long‐lasting immune regulatory functions both *in vivo* and *in vitro* 33. Moreover, the adult MSCs undergoes replicative senescence at a very early stage of proliferative cycle rendering it disadvantageous to use the cells for transplantation 3. Hence, for mainstream therapeutic applications alternate source of MSCs must be considered.

In this regard, human‐induced pluripotent stem cells (hiPSCs) reprogrammed from human adult somatic cells, converge to a better‐defined ground state of pluripotency. Human‐induced pluripotent stem cells can be differentiated into all three germ layer cell types (Ectodermal, Mesodermal and Endodermal) of the organism and – while in the pluripotent state – can be cultured virtually indefinitely without significant signs of replicative senescence. A recent breakthrough in the generation of hiPSCs from human somatic cells by using defined factors, 34, 35 could facilitate generation of patient‐specific iMSCs derived from hiPSCs. The iMSCs have the capabilities for utilization in a broad range of regenerative medical applications. Hence, they are often considered as readily accessible promising source of stem cells for future clinical therapies 3. The iMSCs shared the similar properties compared to the ESC‐derived MSCs 33. Recent studies have also revealed that biomimetic surface results in the rapid and efficient derivation of iMSCs from hiPSCs 36. However, several challenges need to be effectively tackled before iMSCs could be favourably used for translational applications.

## Human pluripotent stem cells (hESCs & hiPSCs) as a novel cell resource for generating clinical‐grade products

The PSCs could serve as an alternate source for the generation of MSCs. Embryonic stem cells can be used as an efficient source to generate the MSCs almost indefinitely, attributed to tremendous proliferation potential of ESCs 37. Nonetheless, ethical concerns, allogenicity and immune reactivity proffer ESC‐derived MSCs unsuitable for clinical applications 30. On the other hand, efficient hiPSCs reprogramming methods could be successfully used to obtain patient‐specific iPSCs. Takahashi and Yamanaka were the first groups to demonstrate that mouse 32 and human 34 somatic cells could be successfully converted to iPSCs through the retroviral delivery of Oct4, Sox2, Klf4 and C‐Myc. Further characterization of iPSCs indicated human iPSCs are similar to human ESCs in their morphology, gene expression profile, *in vitro* differentiation potential and teratoma formation 30. Different types of human somatic cells have been successfully shown to reprogram into hiPSCs (Table 1).

**Table 1:** Different types of human somatic cells that have been reprogrammed to induced pluripotent stem cells (hiPSCs)

Cell source	References
Bone marrow MSCs	38
Adipose tissue‐derived stem cells	39
Cord blood cell	40
Keratinocytes	41
Skin fibroblasts	41
Mammary epithelial cells	42
Renal epithelial cells	43
Corneal epithelial cells	44
Peripheral blood cells	45
Umbilical cord MSCs	46
Placental MSCs	47
Amniotic membrane MSCs	46
Amniotic fluid‐derived cells	48

The discovery of hiPSCs has accelerated the regenerative medical research 19. Human‐induced PSCs are cells that have the capability of differentiating into all somatic cell derivatives (all three germ layers, for example, ectoderm, mesoderm and endoderm) and also, make a contribution to the germline (Table 2 shows a catalogue of different cell types derived from iPSCs); this unique ability of contribution to chimera and indefinite self‐renewal provides a unique opportunity for autologous personalized cell‐based therapy 34, 49, 50. Towards future studies, hiPSCs are considered as the driving force for personalized cell replacement therapy 51.

**Table 2:** List of different cell types including iMSCs derived from hiPSCs

Cell types	References
Ectoderm	
Neural	52
Retinal pigment epithelial cells	53
Corneal epithelial cells	44
Mesoderm	
Cardiomyocytes	54
Adipocytes	55
Osteocytes	56
Chondrocytes	57
iMSCs	58
Haematopoietic stem cells (HSCs)	59
Erythrocytes	60
Platelets	61
Endothelial cells	62
Neutrophils	63
Endoderm	
Lung and airway epithelial cells	64
Nephrogenic intermediates	65
Follicular epithelial cells	66
Hepatocytes	67
Kidney progenitor cells	68, 69
Pancreatic beta cells	70
Germ cells	71, 72

## Establishment of reliable and standardized source of functional MSCs for regenerative applications

The establishment of a reliable source of autologous, transgene‐free progenitor cells have enormous potential in the field of cell‐based regenerative medicine 3, 4, 30, 33, 34, 35, for example, in preparing a therapeutic strategy for infants born with devastating birth defects 73, 74, 75, 76, 77, 78, 79, 80, 81, 82. However, standardization of MSCs remains a major obstacle to the therapeutic usage in regenerative medicine 30, 31, 83. Comparison of experimental data with different studies becomes difficult when starting materials and culture conditions affects cell preparations 6, 8, 10, 16, 17, 18.

Recent studies using RNA‐based technology 84, pluripotency‐associated protein transfection 85, non‐integrating methods of the pluripotent gene containing plasmid usage 86 and a pluripotent gene containing Sendai viral vectors 87, 88 are hinting towards safe clinical usage of footprint‐free hiPSC‐derived cellular products, such as iMSCs, since these directed differentiated cells will not have any risk of undesired genomic modifications associated with reprogramming protocol.

## iMSCs as a novel source of therapeutically active MSCs

The adult MSCs does not exhibit long‐lasting immunoregulatory functions *in vitro* and *in vivo* 10. The primary source of MSCs with high‐proliferation potential has been reported as a suitable alternative to the adult MSCs sources 33. The development of hiPSCs has, in turn, led to the culmination of the unique ability to generate iMSCs by directed differentiation (Table 3). Recent data suggest that iMSCs are emerging as a strong contender for the new sources of MSCs that could be suitable to replace the adult MSCs. Particularly, of late many studies have reported successful derivation of functional MSCs from iPSCs (iMSCs) 33, 36, 50, 58, 89, 90, 91, 92. The iMSCs are a novel class of stem cells that augments effective and reliable regeneration than contemporary methods. The iMSCs can be obtained from the readily accessible adult tissues and exhibit greater proliferation potential than the traditional sources of MSCs 58. Because of the promising pre‐clinical and clinical therapeutic potential of MSCs, the iMSCs derived from iPSCs may serve as an alternate and inexhaustible source 93. Additionally, the synthetic coating has been shown to assist in the derivation of iMSCs. The derivation of iPSCs into iMSCs on synthetic polymer coating, PMEDSAH [Poly [2‐(methacryloyloxy) ethyl dimethyl‐(3‐sulfopropyl) ammonium hydroxide] resulted in high differentiation efficiency, tri‐lineage differentiation potential and expression of characteristic MSCs markers (CD73^+^, CD90^+^, CD105^+^, CD166^+^, CD31^−^, CD34^−^ and CD45^−^) 91. Similarly, Liu *et al*. has shown that iMSCs could be orderly derived in a single step from iPSCs on fibrillar collagen coating 36. In a recent study, Chen *et al*. has shown that treatment of iPSCs with SB431542, a transforming growth factor β pathway inhibitor to generate epithelial monolayer‐like cells in two‐dimensional (2D) culture system followed by induction of epithelial–mesenchymal transition lead to rapid and reliable differentiation into iMSCs 50. Overexpression of Oct4 along with the combination of GSK3 inhibitor has been demonstrated to reprogram CD34^+^ peripheral blood or cord blood into functional mesenchymal stromal cells 94.

**Table 3:** Cell culture supplements that promote *in vitro* derivation of iMSCs from hiPSCs

Materials/Additives	References
Synthetic polymer, PMEDSAH	91
Fibrillar collagen	36
SB431542, a TGF‐β pathway inhibitor	50
RGD (Arg‐Gly‐Asp) peptides	95
Fibronectin (Fn)	95
Fibronectin‐like engineered polymer protein (FEPP)	95
Extracellular matrix, Geltrex	95
Platelet concentrate	95
Oct4	94
CHIR99021, GSK inhibitor	94

## Phenotypic features of iMSCs

The specific cell surface marker on the human MSCs remains to be properly elucidated. Currently, a panel of markers is used to characterize the MSCs isolated from different tissue sources, since there is no specific marker for identifying the bonafide MSCs. The iMSCs satisfies the essential criteria's such as plastic adherence, expression of key MSC surface markers and tri‐lineage differentiation capability properties as laid down by the International Society of Cellular Therapy 3. Himeno *et al*. have demonstrated that iMSCs from mice exhibited characteristic mice MSC surface marker such as CD105, CD140a, Sca‐1 and CD44 as previously described 19, 96. The immunosuppressive, cytoprotection and tissue regeneration properties are exerted by the paracrine factors secreted by the MSCs 97, 98, 99. The iMSCs and ESC‐derived MSCs displayed attenuation of proliferation and cytolytic activity of NK cells in a similar way to BMMSCs. The iMSCs offer vast superiority than traditional sources of MSCs, as they can be generated from any tissue source from the body and theoretically iPSCs pose unlimited growth potential. Thus, iMSCs should serve as an inexhaustible source of MSCs 3. The human MSCs from various tissue sources are typically identified by the expression CD29, CD44, CD73, CD90, CD105, CD146 and CD166. Newer studies have reported that human iMSCs exhibited the above indicated typical characteristics of adult MSCs 33.

## Biological characteristics of iMSCs:

**Figure 1:**
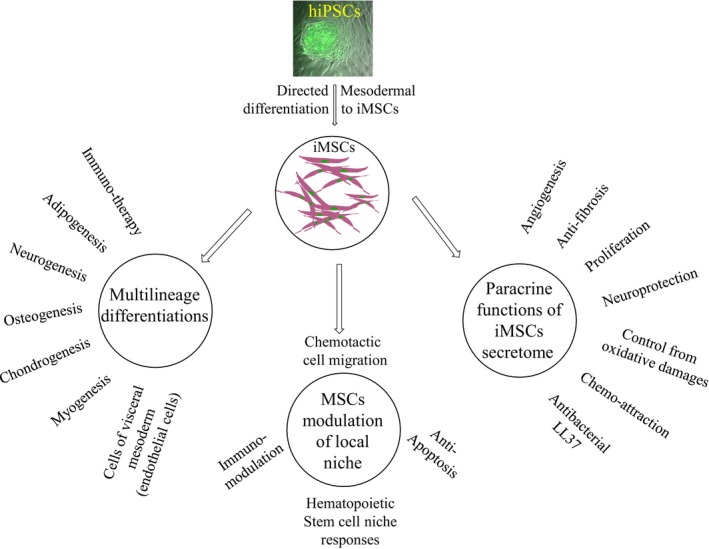
Intrinsic features of iMSCs, which may allow them to have better biological effectiveness compared to adult hMSCs. Multilineage differentiations may obtain a variety of specialized cells for cell replacement therapy (Table 2 lists different types of cells obtained from hiPSCs). Various cytokines in their secretome profile also help immunomodulation, antifibrotic, anti‐apoptotic activities. The microenvironment modulatory paracrine factors may exert a wide range of cellular functions on local cellular niche components *via* the release of the suitable bioactive compound.

The iMSCs and ESC‐derived MSCs displayed similar strong immunosuppressive characteristics 33, also iMSCs display a wide range of cytokine profiles, microenvironment modulatory paracrine factors and exert different functions on the local cellular niche components *via* secretion of suitable bioactive molecules (Fig. 1). Giuliani *et al*. further reported that there was no marked functional variability between iMSCs and ESCs–MSCs 33. Unlike BMMSCs, iMSCs and ESCs–MSCs that could be subjected to long‐term culture without resulting in explicative senescence 33. Studies by Lian *et al*. have shown that iMSCs display typical MSC characteristics and there were no differences between human iMSCs and human ESC‐derived MSCs 58, 83. More robust proliferation was observed in iMSCs than BMMSCs. The iMSCs could be easily scaled up to more than 40 passages while stably maintaining normal diploid karyotype, and consistent gene expression and surface antigen profile 58. Human iMSCs apart from typical MSCs characteristic markers such as CD29, CD44 and CD73 also expressed a higher level of endogenous pluripotency markers such as Oct4 58. Liu has proposed that iMSCs derived from blood cells could be used as a novel and patient‐specific source for usage in disc repair 100. Comparative study of DNA methylation profiles of iMSCs with normal MSCs and PSCs suggested that iMSCs maintained donor‐derived epigenetic differences 101. In a recent study, published by Frobel *et al*. iMSCs are starter MSCs and subjected to epigenetic analysis. The study indicated that morphology, immunophenotype, *in vitro* differentiation and gene expression of iMSCs were consistent with the initial donor MSCs population. Except iMSCs were impaired in suppressing T‐cell proliferation. The iMSCs retained donor‐derived DNA methylation (DNAm) profile. However, tissue‐specific and age‐related DNAm profiles of iMSCs were completely erased. Further, the iMSCs reacquired senescence‐associated DNAm. The study also contrastingly highlights that iMSCs reacquire incomplete immunomodulatory functions 102.

## Directed Differentiation of iMSCs

The use of stem cells and biologically suitable scaffolds offer the full potential for tissue regeneration. Transplantation of lineage‐committed cells can obviate *in vivo* teratoma formation that is caused by the rapid proliferation and uncontrolled spontaneous differentiation of PSCs. Thus, controlled differentiation of hiPSCs into cells that resemble adult MSCs is an attractive approach to obtain a readily available source of progenitor cells for tissue engineering. Unlike previously reported methods that typically rely on the addition of soluble factors to affect PSC differentiation, a recent study by Liu *et al*. reports an alternative approach using a biomaterial coating on a cell culture plate made of fibrillar collagen Type I to promote the derivation of MSC‐like cells. This study has reported a collagen matrix that could potentially play a positive role in regulating the differentiation of hESCs and hiPSCs towards a multipotent mesenchymal progenitor cell 36. Activation of epithelial‐to‐mesenchymal transition (EMT) of epithelial cells has been used successfully by others for generation of MSC‐like cells from hESCs 103. A study by ThienHan *et al*. to generate MSCs from human iPSCs, and investigate the osteogenic differentiation of iMSCs seeded on biofunctionalized CPCs containing RGD (Arg‐Gly‐Asp) peptides, fibronectin (Fn), Fibronectin‐like engineered polymer protein (FEPP), Geltrex and platelet concentrate has also been reported 80. A significant part of the study dedicated to the investigation of iMSCs proliferation and osteogenic differentiation atop calcium phosphate cement (CPC) containing biofunctional agents was also evaluated 80.

## Therapeutic applications of iMSCs

**Figure 2:**
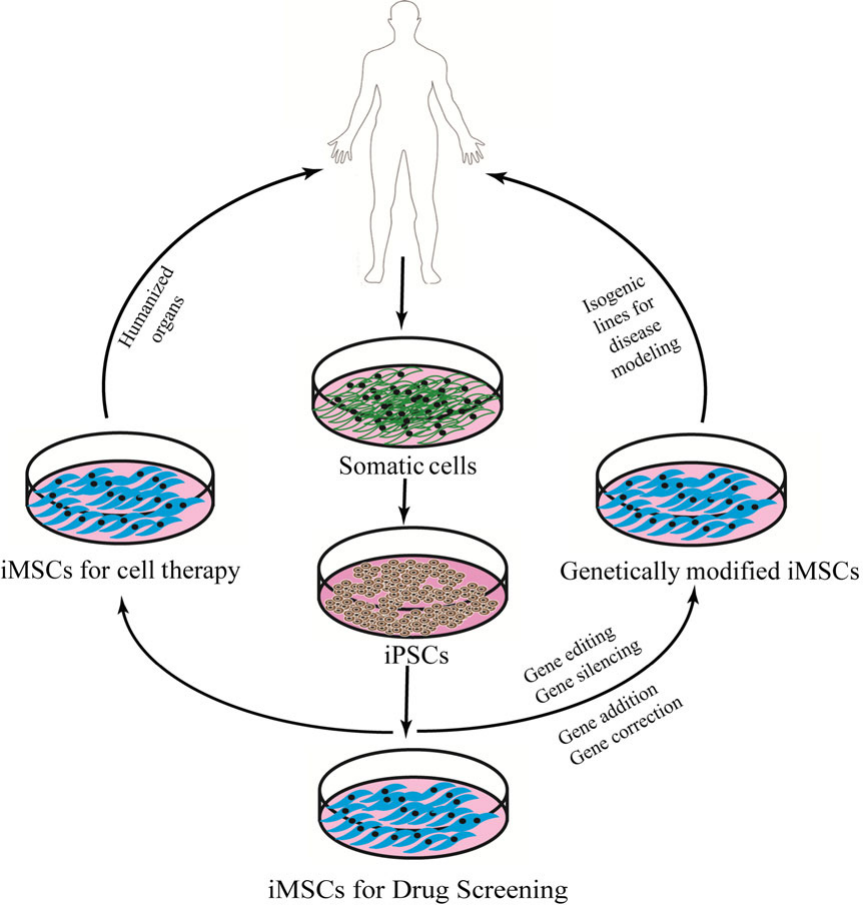
Potential application overview of iMSCs derived from hiPSCs.

The iMSCs could be effectively used for diseases modelling, drug screening and therapeutic applications (Fig. 1). The immunological concerns on cell therapy can be effectively bypassed by iMSCs 51. Nevertheless, the long‐term studies on the immunosuppressive activity of the iMSCs remain to be explored 33. Mesenchymal stem cells are considered as the first line of prophylactic treatment for GVHD and organ transplantation owing to their immunoregulatory properties 27, 104, 105, 106. During allogeneic transplantation, the circulating NK cells target and destroy the graft 107, 108. On the other hand cotransplantation of MSCs prevent GVHD by attenuating the cytotoxic activity of NK Cells 27, 105, 106. Under *in vitro* culture conditions Giuliani *et al*. has shown that human MSCs derived from the iPSCs considerably down‐regulated NK cell cytolytic capabilities 33. The iMSCs were more potent than the BMMSCs. Thus, iMSCs can be graded as a useful therapeutic option to prevent allograft rejection 33. The study from Himeno *et al*. showed MSCs from iPSCs ameliorated diabetic polyneuropathy (DNP) in mice 19. The results suggest that effects of DNP by MSCs might be because of the secretion of angiogenic/neurotrophic factors and differentiation into Schwann type cells. Mesenchymal stem cells have also been reportedly considered as a potential treatment option for periodontal defects arising from periodontitis. A report by Hynes *et al*. indicated that iMSCs facilitated the periodontal regeneration coupled with newly formed mineralized tissue in periodontitis rat models 3. Recently, Yang *et al*. demonstrated that tumour necrosis factor alpha‐stimulated gene‐6 (TSG6) expressing iMSCs were capable of decreasing the inflammation in experimental periodontitis model and inhibiting alveolar bone resorption 109. Human MSCs have emerged as a promising therapeutic source for treating myocardial and limb ischaemia 110, 111. An investigation by Lian *et al*. revealed that human iMSCs attenuate limb ischaemia in mice 58. Further analysis showed that transplantation of iMSCs into mice exhibited better attenuation in hindlimb ischaemia than adult BM‐MSCs. The greater therapeutic efficacy can be attributed to their ability to survive for a longer time after transplantation. Tracking of transplanted iMSCs divulged, iMSCs could engraft and survive for more than 5 weeks following transplantation 58. Wei *et al*. indicated human iMSCs could continuously proliferate for more than 32 passages without undergoing cellular senescence and displayed superior wound healing and pro‐angiogenic properties 92. The iMSCs derived on a synthetic polymeric coating; PMEDSAH resulted in novel bone formation when transplanted into the mice with calvarial defects 91. Zang *et al*. have shown that iMSCs derived from Hutchinson–Gilford Progeria syndrome (HGPS) were helpful in studying the molecular pathology of HGPS 89. In a recent study, Liu *et al*. has successfully utilized iMSCs for modelling Fanconi anaemia. The Fanconi anaemia iPSC‐derived MSCs displayed premature senescence 112. Also, hiPSC‐derived cells have been successfully used to model various other diseases. We have provided the comprehensive details below in Table 4, a list of disease modelling using hiPSCs (Fig. 2).

**Table 4:** Disease modelling using hiPSCs

Disease modelling		References
Neurological		
Development	Fragile X/ataxia syndrome (FXA)	74, 113
	Rett syndrome (RS)	75
	Angleman syndrome	76
	Prader–Willi syndrome	76
	Timothy syndrome (TS)	77
	Microcephaly (MC)	78
	Hereditary spastic paraplegias (HSP)	79
	Olivopontocerebellar atrophy (OPCA)	80
	Pelizaeus–Merzbacher disorder (PMD)	81
	Mitochondrial encephalopathy with lactic acidosis and stroke‐like episodes (MELAS)	82
	Glioblastoma iPSCs	114
	Childhood cerebral adrenoleukodystrophy (CCALD)	115
	Multiple sclerosis	116
	Autism spectrum disorder (ASD)	117
	Cernunnos deficiency syndrome (XLF)	118
	William–Beuren syndrome (WBS)	119
	William–Beuren region duplication syndrome (WBDS)	119
Degenerative	Alzheimer's (AD)	120, 121, 122
	Schizophrenia (SCZD)	123
	Spinal muscular atrophy (SMA)	124
	Parkinson disease (PD)	125, 126, 127
	Huntington disease (HD)	125, 128
	Amyotrophic lateral sclerosis (ALS)	129
	Familial dysautonomia (FD)	130
	X‐linked adrenoleukodystrophy (X‐ALD)	131
	Machado–Joseph disease (MJD)	132
	Friedreich's Ataxia (FRDA)	133
	Familial transthyretin amyloidosis (ATTR)	134
	Tauopathies (TAP)	135
	Diabetic polyneuropathy (DPN)	19
	Gaint axonal neuropathy (GAN)	136
	Menkes disease (MD)	137
	Frontotemporal dementia (FTD)	138, 139
	Spinal cerebral ataxia type2 (SCA2)	140
	Ataxia telangiectasia (AT)	141
	Dravet syndrome (DVS)	142
Hematological	Swachman–Bodian–Diamond syndrome (SBD)	125
	Adenosine deaminase deficiency (ADA) severe combined immunodeficiency (SCID)	125
	Fanconi anemia (FA)	143
	Sickle cell anaemia (SCA)	144
	Beta‐thalassaemia (BT)	145
	Polycythaemia vera (PV)	146
	Congenital amegakaryocytic thrombocytopenia (CAMT)	147
	Paroxysmal nocturnal haemoglobinuria (PNH)	148
	Dyskeratosis congenita (DC)	149
	α‐Thalassaemia (AT)	150
	Aplastic anaemia (AA)	151
	Myeloproliferative disorder (MPN)	152
	Chronic myeloid leukaemia (CML)	153
	Juveline myelomonocytic leukaemia (JMML)	154
	Chronic infantile neurological, cutaneous and articular syndrome (CINCA)	155
	X‐linked chronic granulomatous disease (X‐CGD)	156
	Severe congenital neutropaenia (SCN)	157
	Wiskott–Aldrich syndrome (WAS)	158
Metabolic	Gaucher disease type III (GD)	125
	Juvenile diabetes mellitus (JDM)	125
	Lesch–Nyhan syndrome (LNS)	125
	Aplha1‐Antitrypsin deficiency (A1ATD)	159
	Pompe disease (PomD)	160
	Familial hypercholesterolaemia (FH)	161
	Tyrosinaemia (TYS)	162
	Glycogen storage disease type1 (GSD)	162
	Progressive familial cholestasis (PFD)	162
	Crigler–Najjar syndrome (CN)	162
	Hurler syndrome (HS)	163
	Neuronal ceroid lipofuscinosis (NCL)	164
	Wilson's disease (WD)	165
	Mitochondrial diabetes (MT)	166
	Fabry disease (FD)	87
	Mucopolysaccharidosis type IIIB disease (MPS)	167
Cardiovascular	LEOPARD syndrome (LS)	78
	Long QT syndrome type 1 (LQTS1)	168
	Long QT syndrome type 2 (LQTS2)	169
	Long QT syndrome type 3 (LQTS3)	170
	Supervascular aortic stenosis (SVAS)	171
	Hypertrophic cardiomyopathy (HCM)	172
	Diabetic cardiomyopathy (DCM)	173
	Hypoplastic left heart syndrome (HLHS)	174
	Moyamoya disease (MMD)	175
	Catecholaminergic polymorphic ventricular tachycardia (CPVT)	176
	Familial dilated cardiomyopathy (DCM)	177
	Familial hypertrophic cardiomyopathy (HCM)	178
Primary immunodeficiency	SCID/Leaky SCID	179
	Omenn syndrome (OS)	179
	Cartilage–hair hypoplasia (CHH)	179
	Herpes simplex encephalitis (HSE)	179
Musculoskeletal disorder	Craniometaphyseal dysplasia (CMD)	88
	Duchenne muscular dystrophy (DMD)	125
	Becker muscular dystrophy (BMD)	125
	Osteogenesis imperfect (OI)	180
	Thanatophoric dysplasia (THD)	181
	Achondroplasia (ACH)	181
	Hutchinson–Gilford progeria syndrome (HGPS)	182
	Werner syndrome (WS)	183
	Facioscapulohumeral muscular dystrophy (FSHD)	184
	Limb‐girdle muscular dystrophy (LGMD)	185
	Myotonic dystrophy type 1 (MyD1)	186
	Marfan syndrome (MFS)	187
	Fibrodysplsia ossificans progressiva (FOP)	188
Lung disorder	Cystic fibrosis (CF)	189
	Pulmonary alveolar proteinosis (PAP)	190
	Emphysema (EP)	191
Dermatological Disorder	Recessive dystrophic epidermolysis bullosa (RDEB)	192, 193
	Scleroderma (SC)	191
	Focal dermal hypoplasia (FDH)	194
	Hermansky–Pudlak syndrome (HPS)	195
	Chediak–Higashi syndrome (CHS)	195
Cancer	Breast cancer (BC)	196
Opthalmological disorder	Retinitis pigmentosa (RP)	53, 197, 198
	Gyrate atrophy (GA)	199
	Best disease (BD)	200
	Cataract (Cat)	201
	Ectrodactyly‐ectodermal dysplasia‐cleft syndrome (EEC)	202
Nephrology	End stage renal disease (ESRD)	203
Aneuploidy	Turner syndrome (TS)	204
	Warkany syndrome (WKS)	204
	Patau syndrome (PS)	204
	Emanuel syndrome (ES)	204
	Klinefelter's syndrome (KS)	205
	Down's syndrome	125

## Recent developments in safe clinical products

### Challenges & strategy to overcome them for Clinically Relevant iMSCs

Safety and Efficacy of iMSCs are of paramount importance to succeed in the field of translational regenerative medicine. The viral vector‐based strategy for reprogramming might result in tumour formation as a result of insertional mutagenesis of the transgene. C‐Myc is a proto‐oncogene that has been shown to increase the efficiency of reprogramming by suppressing the tumour suppressor p‐53 gene. The overexpression of proto‐oncogene and moderating of tumour suppressor genes render hiPSCs beneficial results regarding higher proliferative advantage for downstream translational applications 206. Consequently, many studies have unveiled several different strategies for generation of safer iPSCs. In 2010, Yamanaka's group suggested using L‐Myc as an alternative to C‐Myc for reprogramming based on the result that L‐Myc maintained in the reprogramming efficiency without inducing any tumorigenesis 206. Fang *et al*., reported generated iPSCs devoid of C‐Myc enervated retinal ischaemia and reperfusion injury following transplantation in rat models 207. The starter cell type has an enormous impact on reprogramming, differentiation and *in vivo* functionality because of epigenetic memory. Until now, there are no data on the best starter cell type for a particular clinical application. Hence, more research needs to be conducted to determine suitable starter cell type based on the type of clinical application 30. While iPSC‐derived cell source are emerging as a replacement cell source, their traits of self‐renewal and pluripotency after *in vivo* transplantation often leads to tumorigenicity and genomic instability might result in low clinical utility 3. The nature of pluripotency transgene elements present in the iMSCs is arduous to predict. Hence, iMSCs have been thoroughly characterized for silencing of the transgene expression and safe transgene integration 33. Presently, only initial studies are reported on preclinical applications of iMSCs. Hence, long‐term, multicentric, pre‐clinical and clinical studies are required for accurate prediction of iMSCs for the translational purpose 33. The recent development of non‐viral‐based generation of iPSCs might pave the way for considering iPSCs as a suitable candidate for biotherapeutics 86, 208, 209, 210. Newer technologies without viral transgene such as chemicals, plasmids and recombinant protein‐based approaches might augment the clinical utilization of these safe iPSCs 85, 211, 212. The low efficiency of iPSCs generation might be a serve‐debilitating factor to consider iPSCs/iMSCs for translational applications. Hence, more research needs to be focused on scaling and optimizing the quality of iPSCs 58.

### Regulatory issues for future safe therapies using hiPSCs

The iPSCs present unique sets of technical and regulatory hurdles when compared to even ESCs for translational applications 213. The issues regarding the cell and gene therapy in every country are governed by its sovereign regulatory body. In the United States, the human iPSC products are regulated by Centre for Biologics Evaluation and Research at the United States Food and Drug Administration (USFDA) 214. Before proceeding with the clinical trials, the iPSC‐derived products are subjected to preclinical testing that requires extensive examination of safety, feasibility and efficacy 215. Pre‐clinical studies involve a comparative analysis of the various parameters between healthy animals and disease models. According to the FDA guidelines, the same cells used in preclinical trials should be used during clinical trials 215. Small animal models, such as rodents are used in preclinical studies. However, rodent models, although could be used for basic biological studies have a poor predictive outcome in term of clinical efficacy 215. Consequently, pre‐clinical studies consisting of large animal models such as swine, primates, *etc*., are favourable as they have relatively longer life span and displays physiological similarities to humans albeit a limited number of disease models and inability to modify the genome with ease constitute major road block in the usage of large animal models 215. For a particular disease condition, a single satisfactory model is not present. Hence, pre‐clinical testing must be carried out using suitable alternative models to highlight potential limitations and assist in finding suitable alternative avenues for handling the disorder 215. Necessary precautions must be undertaken before extrapolating the results from animal models to clinical trials 215.

Efficacy of the transplanted cells *in vivo* is not well documented. A few studies have demonstrated that transplantation of PSCs and differentiated cells resulted in poor survival of the cells 216, 217, 218, 219. The fate of the transplanted cells must be evaluated to ascertain the tangible effectiveness of the cells *in vivo* following transplantation. Hence, suitable surgical/imaging techniques should be developed for *in vivo* fate mapping of the cells 215.

Current good manufacturing protocol guidelines must be followed to generate and characterize iPSC‐based products 215 for any future clinical applications. The quality of cell products and homogeneity of the cell population will determine the effect, risk and potency of the iPSC‐based therapy 215. Method and duration of storage, viability, cell line contamination and risks of transmissible infections are some of the other possible confounding factors that can affect the cell therapy 215. Before scaling up towards clinical trials questions such as ideal cell source, efficient reprogramming and differentiation protocols, demonstration of safety and functionality have to be addressed 215.

Generation of iPSCs from somatic cells requires a significant amount of molecular manipulations 213 either by viral vectors containing reprogramming genes 34 or transfection of reprogramming mRNAs 84 or purified reprogramming factors 85 or transfection by non‐viral vectors containing reprogramming gene methods 86. The viral‐based reprogramming strategies form the basis of added concerns because of random integration into the host genome 34. On the contrary use of retroviral‐based genetically modified cells is technically permitted for human clinical trials under the existing National Institutes of Health (NIH), guidelines 213. Recent strategies for using small molecule‐based reprogramming & differentiation must be explored to develop and differentiate into clinical relevant cell types 220. Generation of iPSCs require a significant amount of manipulations 213. The viral‐based reprogramming strategies form the basis of added concerns because of random integration into the host genome 34. On the contrary, use of retroviral‐based genetically modified cells is technically permitted for human clinical trials under the existing NIH, guidelines 213. Recent strategies for using small molecule‐based reprogramming & differentiation must be explored to develop and differentiate into clinical relevant cell types 220. Every iPSC line would exhibit unique genetic and epigenetic constitution. Hence, each and every cell line has to be subjected to independent characterization to determine its precise characteristic features 213. It is necessary to determine to what extent iPSCs are similar to ESCs. Besides safety, efficacy, stability, heritability and absence of biased lineage differentiation have to examine and documented 213.

Immune response against transplanted cells presents a critical challenge that can detrimentally affect the outcome of therapy. Some of the important questions as to why the donor cells pose a risk of immune response or genetic diseases, cell efficiency, cells exhibiting risk of contamination, effectiveness and safety of transplanted cells has to be answered, if they are perceived to develop into potential therapeutic agents 221, 222, 223. Tumour formation remains one of the most important concerns while using the pluripotent cells or PSC‐derived products. It has been well documented that the reminiscent PSCs present in differentiated cells could effectively give rise to tumour formation 217, 219, 224, 225, 226, 227, 228.

One of the biggest advantages of iPSCs is the possibility of generation of patient‐specific autologous cell lines. Hence, the cumbersome procedure of screening against different cell lines for a proper match is excluded 34, 213, 229. The method of selection and characterization criteria needs good manufacturing protocol 213. The combination of a proven gene therapy with proven PSC‐derived products might hold a great potential for therapeutic application albeit certain technical and regulatory hurdles. Hence, suitable regulatory guidelines should be established for the application of genetically modified stem cells 213.

## Conclusion

The invention of cellular reprogramming of adult cells from the terminally differentiated state of PSCs state with the help of transcription factors, biological factors and small molecules open up a large window of opportunity in the field of regenerative medicine. By incorporating the advantages of both iPSCs and MSCs, the resulting iMSCs are emerging as a novel stem cell population 3. The iMSCs generated from iPSCs successfully exhibited all the fundamental criteria for defining the MSC population based on the existing knowledge 3. Data indicate that iMSCs can be used as a promising alternative strategy for treatment of various immune‐mediated diseases 33. Although, the concept of iMSCs is at its nascent stage, recent studies nevertheless provide the proof of concept that functional iMSCs could be successfully generated from iPSCs that exhibit robust proliferation and differentiation potential, which could be used for tissue repair and engineering applications 58. The development of iMSCs offers promise of patient‐specific, cost‐efficient and batch to batch consistency 58. Presently, the scope of iMSCs is limited to the pre‐clinical utility for tissue engineering‐based treatment approaches. Further pre‐clinical and clinical studies are required before scaling it towards routine clinical utility.

## Conflicts of interest

None.
